# Carboplatin, gemcitabine, and mifepristone for advanced breast and recurrent/persistent epithelial ovarian cancer

**DOI:** 10.1007/s10549-025-07783-7

**Published:** 2025-08-08

**Authors:** Erica M. Stringer-Reasor, Poornima Saha, Masha Kocherginsky, Ricardo Lastra, Gabrielle Baker, Ernst Lenygel, Theodore Karrison, Lauren Olalde, Elizabeth Mokrzycki, Olwen M. Hahn, Philip C. Hoffman, Suzanne D. Conzen, Gini F. Fleming, Rita Nanda

**Affiliations:** 1 Department of Medicine, Section of Hematology/Oncology, University of Alabama at Birmingham, 1720 2nd Avenue South, NP 2510, Birmingham, AL 35294-3300 USA; 2 Department of Medicine, Endeavor Health, Evanston, IL USA; 3 Department of Preventive Medicine, Northwestern University Feinberg School of Medicine Chicago, Chicago, IL USA; 4 Department of Pathology, The University of Chicago, Chicago, IL USA; 5 Department of Pathology, Beth Israel Deaconess Medical Center, Boston, MA USA; 6 Department of Obstetrics and Gynecology, Section of Gynecologic Oncology, University of Chicago, Chicago, IL USA; 7 Department of Medicine, Section of Hematology/Oncology, University of Texas Southwestern Medical Center, Dallas, TX USA; 8 Department of Medicine, Section of Hematology/Oncology, The University of Chicago Medicine, Chicago, IL USA

**Keywords:** Mifepristone, Breast cancer, Ovarian cancer, Carboplatin, Gemcitabine

## Abstract

**Purpose:**

Preclinical models of glucocorticoid receptor (GR)-positive breast cancer (BC) and ovarian cancer (OC) suggest GR activity inhibits chemotherapy-induced apoptosis, and GR antagonism using mifepristone (Mif) enhances cytotoxicity. We performed a phase I trial combining mifepristone, carboplatin (C), gemcitabine (G).

**Methods:**

A standard “3 + 3” dose escalation scheme was used. Objectives were safety and to determine the maximum tolerated dose (MTD) of Mif + CG. CG was administered intravenously on days 1 and 8 of a 21-day cycle, and mifepristone was administered orally the day before and day of chemotherapy.

**Results:**

Thirty-one patients enrolled with a median age of 54 years; the median prior metastatic regimens were one. Twenty-five patients were evaluable for dose-limiting toxicities (DLT) including 16 BC and 9 OC. Dose was de-escalated to dose level (DL) -1 due to 2/4 neutropenia-related DLT's. DLT definition was updated to exclude hematologic DLTs starting at DL-1. The dose  was further de-escalated due to neutropenia, and 2/3, 1/4 and 0/6 patients experienced a DLT at DL-1, DL-2, and DL-3, respectively. At DL-1, prophylactic pegylated granulocyte colony-stimulating factor (G-CSF) was instituted. Dose levels -1 and -2 were expanded to add 3 and 6 patients, respectively, to evaluate tolerability  in dose levels -1a and -2a. There were 3 major responses (1CR, 2PR) at DL1, and 1 CR at DL-1. No responses were observed at lower levels.

**Conclusion:**

The MTD was carboplatin AUC 2  + gemcitabine 600 mg/m^2^ on D1 and 8 with Mif 300 mg D-1 and D1 with pegylated G-CSF administered on day 9 of a 21-day cycle.

## Introduction

While many patients with advanced breast cancer (BC) or recurrent ovarian cancer (OC) experience an objective response to initial therapy, progression of disease and chemotherapy resistance are inevitable. Improved therapies are needed to overcome chemotherapy resistance and improve patient outcomes.

Glucocorticoid receptor (GR) is expressed in a significant proportion of breast [1] and ovarian cancers [2, 3]; 32%–94% in breast [4–6] and 38%–88% in ovarian cancers [7, 8]. In vitro and in vivo data suggest that activation of GR in OC and estrogen receptor (ER)-negative breast [1, 9] cancer cells initiate transcriptional cell survival gene expression pathways leading to chemotherapy resistance [3]. These anti-apoptotic signaling pathways can be reversed by GR antagonism [10]. Mifepristone is a highly potent steroidal glucocorticoid receptor (GR) and progesterone receptor (PR) antagonist as well as a moderately potent androgen receptor (AR) antagonist that is not currently approved for the treatment of cancer [11, 12].

Our group previously performed a phase I trial of Mif in combination with nab-paclitaxel for the treatment of patients with advanced, unresectable BC [13]. Up to four prior cytotoxic regimens were permitted. Several significant responses in patients with triple-negative, GR-positive, and taxane-pretreated tumors were observed. However, the nab-paclitaxel levels were variable, and we hypothesized that this variability may be related to pharmacokinetic-pharmacodynamic (PKPD) interactions between nab-paclitaxel and Mif that vary between individuals based on pharmacogenomic differences in cytochrome (CYP) enzyme-dependent metabolism [13].

We therefore selected an alternative chemotherapy regimen unlikely to result in drug-drug interactions (DDI) due to a lack of CYP-mediated metabolism of the chemotherapy agents. Carboplatin (C) and gemcitabine (G) is an effective, well-tolerated chemotherapy regimen for both metastatic BC and OC [14–17]. In addition, neither drug is metabolized to any significant extent by the cytochrome P450 (CYP450) enzyme. Furthermore, corticosteroids, although often used as part of anti-emetic premedication, are not required with the regimen. We hypothesized that GR transcriptional inhibition following Mif (administered prior to chemotherapy and C + G) would enhance cytotoxicity through blocking expression of tumor cell GR-mediated cell survival and chemotherapy-resistance gene expression pathways. The primary purpose of this study was to identify the RP2D for the combination of Mif + C + G therapy.

## Materials and methods

This study was conducted at The University of Chicago Medicine (Chicago, IL) and Endeavor Health (Evanston, IL) and was approved by the respective Institutional Review Boards. Written informed consent from patients was obtained prior to any study procedure. The study was registered on ClinicalTrials.gov as NCT02046421.

### Eligibility criteria

Eligibility requirements included metastatic or locally advanced, unresectable BC or advanced recurrent or persistent epithelial OC, age > 18 years, ECOG Scale of Performance Status (PS) of 0–2, and RECIST measurable disease. Patients with ER and/or PR positive BC had to have progressed on at least one prior hormonal therapy. Patients were required to have adequate organ and marrow function as defined by the study protocol.

### Exclusion criteria

Patients were excluded from this study if they had a history of hypersensitivity to mifepristone, carboplatin, gemcitabine, or drugs of similar chemical composition, or any prior Mif for anticancer therapy. Patients on recent or ongoing corticosteroid therapy were excluded. Patient with OC with platinum refractory disease or prior G therapy were not eligible. Also, patients with BC who had received prior platinum-based therapies and/or G for metastatic disease and BC patients with HER2-positive disease were excluded.

### Drug supply

C and G were obtained commercially. Mifepristone was supplied by Corcept Therapeutics.

### Study treatment

Treatment was administered on an outpatient basis. No concomitant investigational or commercial agents or therapies administered with the intent to treat the patient's malignancy were allowed, except for bisphosphonates (e.g., zoledronic acid) and RANKL inhibitors (e.g., denosumab) for patients with bone metastases.

All patients received C and G along with Mif. Patients were treated with intravenous C and G on days 1 and 8 of each 21-day cycle at a starting dose of C AUC 2 and G 1,000 mg/m^2^ administered over 30 min, respectively. Mif was administered orally with food at a dose of 300 mg per day for two consecutive days, the day prior to (D-1) and the day of chemotherapy infusion (D1). All patients who had received prior C-containing regimens received premedication, such as diphenhydramine 25–50 mg IV or PO and famotidine 20 mg IV or PO 30 min prior to C administration to prevent severe hypersensitivity reactions. No premedication with steroids (e.g., dexamethasone, prednisone, or methylprednisolone) were allowed. Steroids were allowed emergently, such as in the case of C reaction. Anti-emetics such as ondansetron, prochlorperazine maleate, or lorazepam, before, during, or after chemotherapy were allowed. Treatment with all three drugs continued indefinitely until toxicity or progression.

### Dose escalation/de-escalation

A phase I design was utilized with the traditional ‘3 + 3’ design with two cohorts of Mifepristone initially planned (300 mg, 600 mg) to determine the maximal tolerated dose (MTD) when combined with C and G. The starting dose of Mifepristone was 300 mg. Toxicity was assessed on days 1 and 8 during the first cycle of therapy. Adverse events were assessed among all patients for the purpose of dose escalation decisions. The chemotherapy starting dose was C AUC 2 and G 1000 mg/m^2^ (dose level 1), with the plan to deescalate to carboplatin AUC 2/gemcitabine 800 mg/^2^ (dose level -1), C AUC 2/G 600 mg/m^2^ (dose level -2), and C AUC 2/G 400 mg/m^2^ (dose level -3) for subsequent dose levels as needed. Two dose levels were expanded for further evaluation of safety and efficacy including C AUC 2 and G 800 mg/m^2^ (dose level -1a) and C AUC 2 and G 600 mg/m^2^ (dose level -2a) with mifepristone at 300 mg. Mifepristone was not increased to dose 600 mg given the higher than expected neutropenia rates observed with the combination of Mif 300 mg, C, and G.

### Dose-limiting toxicity

Dose-limiting toxicity (DLT) was defined as a grade 3 or higher toxicity occurring during cycle 1, considered at least possibly related to study drugs, that didn’t resolve within 72 h, with the following exceptions: grade 4 neutropenia that persisted for < 5 consecutive days without growth factor support and without febrile neutropenia or infection; grade 3 thrombocytopenia with no evidence of bleeding; grade 3 anemia; grade 3 nausea or vomiting that resolved to grade 1 or less within 3 days; and grade 3 rash or allergic reaction that resolved to < grade 1 within 3 days. Toxicity was graded according to CTCAE version 4.0. A patient who received any amount of drug was considered evaluable for toxicity. However, patients who did not receive at least 4 doses of M and 2 planned doses of C + G during cycle 1 for reasons other than toxicity/tolerability, were considered not evaluable for determination of MTD and were replaced. A dose reduction, omission, or delay of dose for toxicity performed during cycle 1 and cycle 2 day 1 constituted a DLT.

### Study assessments

A physical examination, ECOG Performance Status assessment, complete blood count with differential, and a comprehensive metabolic panel were conducted at baseline and weekly for two weeks in a row with one week off during cycles 1 and 2. With cycle 3 and beyond, study assessments were done on day 1 of each cycle. All adverse events and laboratory abnormalities were assessed at baseline and during treatment. Radiologic tumor measurements were obtained every 2 cycles (6 weeks) for the first 4 cycles, then the frequency of radiologic evaluations was at the discretion of the treating physician.

### Correlative studies

Unstained Sects. (3–5 microns in thickness) of primary tumor, metastatic tumor, or both were requested for each study-enrolled patient. Archival tumor tissue was evaluated retrospectively via immunohistochemistry (IHC) for nuclear receptor expression of GR and AR. For determination of GR expression, anti-GR XP antibody (Cell Signaling, D8H2, 1:40 dilution) was used, and for determination of AR expression, anti-AR antibody (DAKO, AR441, 1:300 dilution) was used. As mifepristone is a strong GR and PR antagonist, but also a moderate AR antagonist, we performed an exploratory correlation of the expression of both GR and AR to response. Tumors were given a score for GR or AR % positivity: 0 expression = score of 0; 1–10% expression = score of 1; 11–25% expression = score of 2; 25–50% expression = score of 3; > 50% expression = score of 4. Tumors with a score of 0, 1, and 2 were considered to have low expression. Tumors with scores of 3 or 4 were considered to have high expression.

### Statistical analysis

This was a phase I clinical trial to determine the MTD, the recommended phase II dose, and DLTs associated with combination treatment of C + G with Mif in patients with advanced BC or recurrent OC. Dose escalation proceeded according to a standard “3 + 3” design with a dose expansion cohort to further assess safety and activity data. There was a predetermined high probability (91%) of dose escalation if the true DLT rate was low (10%), and a low probability (8%) if the true DLT rate was high (60%). All patients who received treatment were evaluated for toxicity. Among 16 patients treated at the MTD, the exact Clopper-Pearson 90% CI for response would be no wider than ± 22%.

Tumor samples from primary and metastatic sites were requested for all patients enrolled on this trial. Immunohistochemical detection of GR was scored and dichotomized (GR + and GR−) as previously described [5]. In addition, all tissues were evaluated for ER, PR, and AR by IHC to evaluate presence or evolution/change of ER, PR, and AR between primary and metastatic tumor. The association between GR expression and treatment response was examined descriptively. Due to the small sample size these analyses were exploratory.

## Results

### Patient characteristics

A total of 31 women were enrolled between December 2013 through October 2016 and 25 were evaluable for response. Eighteen patients had BC and thirteen patients had OC (Table 1). Within the BC cohort, 3 patients had ER/PR positive (ER > 10% and/or PR > 10%) disease and 15 had triple-negative (ER < 10%, PR < 10%, HER2 negative by ASCO CAP guidelines) disease. The median age of participants was 54 years (range 32–76). Twenty-one patients were White, five patients were Black, three were Asian and two were Hispanic. The median number of prior chemotherapies in the metastatic setting was 1 (range 0–4). All patients had received prior anthracycline and/or taxane containing regimens. The three BC patients who had ER/PR positive disease had progressed on prior endocrine therapy.

**Table 1:** Patient characteristics for breast and ovarian cancer

Breast cancer	
Characteristic	N = 18
Age, mean (range) years	51 (32–75)
Female	18 (100%)
Subtype	
ER +	3 (17%)
TNBC	15 (83%)
Race/Ethnicity	
White	12 (67%)
Black/African American	3 (17%)
Asian	2 (11%)
Hispanic	1 (5%)
No. prior therapies metastatic disease	
0	11
1	3
2	4

Ovarian cancer	
Characteristic	N = 13
Age, mean (range) years	59 (41–76)
Female	13 (100%)
Subtype	
High-grade serous	13 (100%)
Platinum sensitive	11 (85%)
Platinum-resistant	2 (15%)
Race/Ethnicity	
White	9 (69%)
Black/African American	2 (15%)
Asian	1 (8%)
Hispanic	1 (8%)
No. prior therapies metastatic disease	
0	1
1	7
2	5

### DLTs

Four patients were treated at dose level 1 with C AUC 2, G 1000 mg/m^2^, and Mif 300 mg (Table 2). Patient 3 experienced grade 3 dose-limiting neutropenia and ultimately progressed after two cycles of therapy. Patient 4 experienced grade 4 neutropenia with cycle 1 requiring dose reduction, but ultimately went on to receive 6 cycles and then by choice had a chemotherapy holiday. She had a CR and remained free of disease as of July 2021 with no further therapy.

**Table 2:** Overall response rates in the breast cancer and ovarian cancer cohorts

Dose level	N. Eval	N. DLT	G2/3 hem tox	Uneval	Best response	Unevaluable
1 (G 1000 mg/m^2^)	4	2	3	0	1CR, 2PR, 1PD; 3/4 ORR	
− 1 (G 800 mg/m^2^)	3	1	2	0	1CR, 2SD; 1/3 ORR	
− 2 (G 600 mg/m^2^)	4	1	3	4	2SD, 1PD, 1NE	1 pneumonia missed Mif dose; 1 bowel obstruction; 1 pt withdrew; 1 missed dose Mif C1D7
− 3 (G 400 mg/m^2^)	6	0	3	1	4 SD, 2 PD	1 missed dose Mif C1D7
− 1a* (G 800 mg/m^2^), expanded	3	1	1	0	2 SD, 1 PD	1 G3 neutropenia C1D8
− 2a* (G 600 mg/m^2^), expanded	6	0	1	1	3 SD, 3 PD	Skipped C1D8 and C2D1 due to neutropenia and gem reduced to 400

*CR* complete response, *PR* partial response, *SD* stable disease, *PD* progressive disease, *NE* not evaluable, *DLT* dose-limiting toxicity, *N* number

^*^Carboplatin AUC 2 was the starting dose at each dose level combined with gemcitabine (G) and Mifepristone (mif) 300 mg the day before and day of treatment

^*^Prophylactic G-CSF administered on day 9

Because two DLTs were observed at dose level 1, the starting dose of G was reduced from 1000 mg/m^2^ to 800 mg/m^2^ for dose level -1. The Mif dose was continued at 300 mg/day the day prior to and the morning of chemotherapy administration. Due to the severe neutropenia observed in dose level 1, growth factor support was initiated for dose level -1 and beyond. At dose level -1, 3 patients were enrolled. One patient had a DLT due to grade 3 elevation in liver function tests requiring dose reduction. Additionally, 2 patients had grade 2/3 neutropenia.

Four patients were enrolled at dose level -2 with a further reduction in G to 600 mg/m^2^. C was continued at AUC 2 and Mif dose was continued at 300 mg/day the day prior to and morning of chemotherapy. Four patients were evaluable for DLT. At dose level -2, there was 1 DLT noted (grade 3 rash) and three patients with grade 2 or 3 neutropenia. Patient 13, who was noted to have grade 3 neutropenia on cycle 1 day 8, ultimately continued therapy with dose reduction of C to AUC 1.6 and G 400 mg/m^2^ for 11 cycles with stable disease, before coming off study for functional decline.

At the next dose level -3, G was further reduced to 400 mg/m^2^. C and Mif were continued at the same doses. There were 6 patients enrolled at this dose level without any DLTs (0/6).

Given the persistent neutropenia thought to be due to CG, the protocol was amended to allow the prophylactic administration of G-CSF on day 9 for subsequent dose levels. Thus, for dose level -1a, G was given at 800 mg/m^2^ with C AUC 2 and Mif 300 mg the day prior to and day of chemotherapy administration. There were 3 patients enrolled at this dose level and one patient had grade 3 dose-limiting neutropenia with treatment delay in C1D8 despite administration of G-CSF.

Six patients were then enrolled at dose level -2a with G dose reduced to 600 mg/m^2^. C remained at AUC 2 and Mif dose remained 300 mg per day the day prior and day of chemotherapy administration. Prophylactic G-CSF was administered on day 9. At this dose level, only 1 patient experienced a DLT of neutropenia. The recommended phase 2 dose was determined to be C AUC 2, G 600 mg/m^2^, and Mif 300 mg per day (day prior to and day of chemotherapy) with prophylactic pegylated G-CSF administered on day 9.

Common grade 1–2 toxicities observed on the study included fatigue, nausea, vomiting, constipation, anorexia, rash, alopecia, and sensory neuropathy. Grade 1–2 anemia and thrombocytopenia were also seen.

### Overall response and tumor characteristics

Overall, thirty-one patients were enrolled in this study and 25 patients were evaluable for response. Two patients had CR, 2 PR, 13 SD, and 8 PD. The patient with breast cancer who achieved CR had tumors expressing < 1% of ER and PR. Of note, patient number 4 in dose level 1 with OC remains in a prolonged CR at > 63 months after six cycles of combined therapy prior to taking a chemotherapy holiday (Tables 2 and 3).

In the breast cancer cohort, 8/18 had high GR (staining 3 or 4) expression. Varying levels of AR expression were observed. Two patients had tumors staining high for both GR and AR. Of the BC patients with high tumor GR expression, three had SD (Table 4).

**Table 3:** Ovarian cancer cohort by response, GR status, AR status, and intensity by immunohistochemical staining

Patient #	Dose level (G, mg/m^2^)	Best response	GR	GR intensity	AR	AR intensity
4*	1000	CR	4	S	3	W
16	600	SD	4	S	3	S
18	400	SD	4	W	2	W
19	400	SD	4	S	1	W
21	400	SD	2	W	1	S
22	400	SD	4	S	0	NA
24	800	SD	4	S	2	S
27	600	SD	4	S	2	W
10	600	PD	3	S	1	W
26	800	PD	4	S	1	W
33	600	PD	4	S	1	W
9	600	NA	4	W	2	W
11	600	NA	4	S	2	W

*GR* Glucocorticoid receptor, *AR* androgen receptor, *S* strong, *W* weak, *SD* stable disease, *PD* progressive disease, *NA* not applicable

^*^Patient number 4 remains in a prolonged CR at > 63 months

**Fig. 1:**
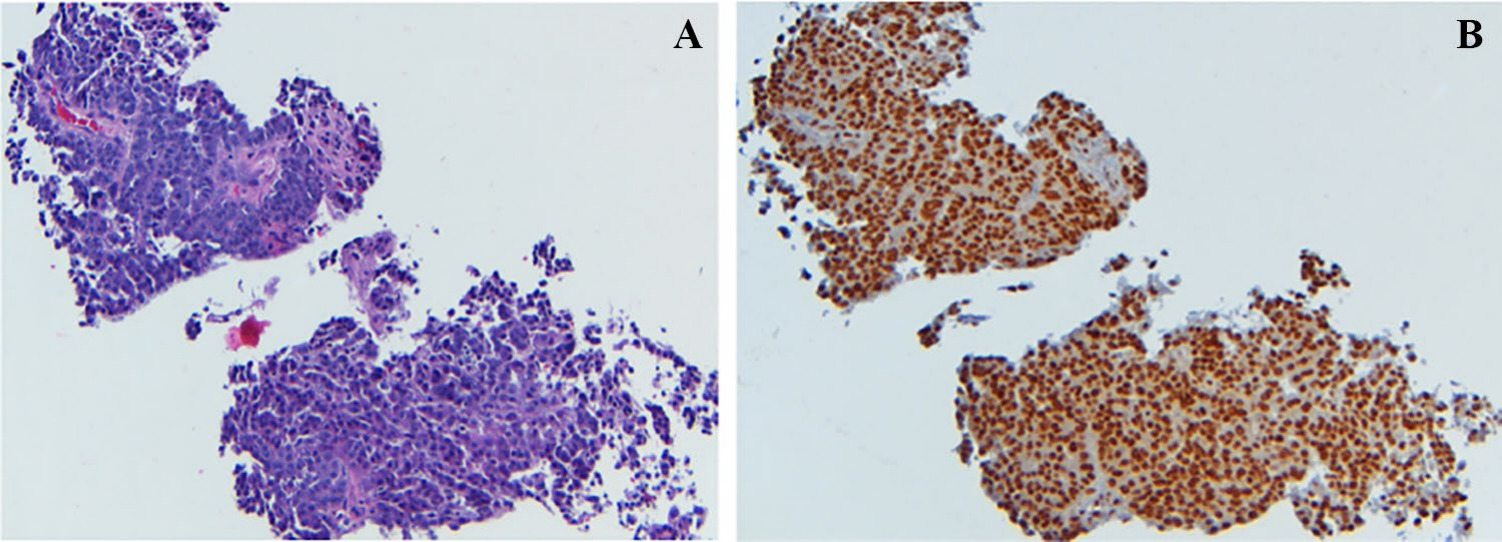
Patient in the ovarian cancer cohort. **A.** H&E stain: High-grade serous ovarian carcinoma **B.** GR strongly positive IHC staining (4 + , strong intensity)

In the OC cohort, 12/13 had high GR expression (staining 3 or 4) by IHC. Two tumors in the OC cohort had both high AR expression by IHC and high GR expression (Table 3). Among the OC patients with high GR expression, one patient had CR at DL1 and 6 had SD. Figure 1 demonstrates a patient with high-grade serous ovarian carcinoma with platinum-resistant disease. The tumor cells strongly expressed > 95% GR and the patient experienced a clinical response after 2 cycles of therapy.

**Table 4:** Breast cancer cohort by response, GR status, AR status, and intensity by immunohistochemical staining

Patient #	Dose Level (G, mg/m^2^)	Best Response	GR	GR intensity	AR	AR intensity
5	800	CR	2	W	0	NA
1	1000	PR	2	W	0	NA
2	1000	PR	1	W	3	S
6	800	SD	1	W	1	W
7	800	SD	4	S	4	S
13	600	SD	2	W	1	W
14	600	SD	3	W	0	NA
17	400	SD	4	W	4	S
25	800	SD	1	W	4	W
29	600	SD	1	W	1	W
30	600	SD	1	W	0	NA
3	1000	PD	2	W	0	NA
20	400	PD	3	W	2	W
23	400	PD	3	W	0	NA
28	600	PD	3	W	0	NA
31	600	PD	4	S	0	NA
12	600	NA *pt off study after C1 due to choice	NA	NA	NA	NA
15	600	NA	4	W	3	W

^*^G gemcitabine, *GR* glucocorticoid receptor, *AR* androgen receptor, *SD* stable disease, *PD* progressive disease, *W* weak, *S* strong, *NA* not applicable

## Discussion

The rationale for the current study design was generated from findings of a previous phase I study of Mif treatment prior to nab-paclitaxel. Mif was given the day prior to and morning of nab-paclitaxel infusion, which was administered on days 1, 8, and 15 of a 28-day cycle. Both dose levels tested were tolerable, however G-CSF was required in some patients due to protocol-defined neutropenia. Subsequently, we concluded that there was a potential drug-drug interaction between nab-paclitaxel and Mif as they are both metabolized through cytochrome P450 liver enzymes CYP3A4 and CYP2C8 leading to higher exposure of nab-paclitaxel. Therefore, for this study the chemotherapy backbone was changed to C and G. This combination has shown activity in the treatment of BC and OC with no appreciable metabolism through the CYP enzymes. This chemotherapy choice alleviated the need to perform PK studies.

In this phase 1 trial the MTD of the Mif with C + G combination was reached at G dosed to 600 mg/m^2^, C remaining at AUC 2, and Mif remaining at 300 mg per day orally administered the day prior and day of chemotherapy with this being determined as the MTD. The most common DLT observed was neutropenia, which was managed with G-CSF prophylaxis. Non-hematological toxicities were mild to moderate in severity, suggesting that treatment with the combination is feasible. The most common treatment-related adverse events were those expected from each agent independently, including fatigue, nausea, vomiting, diarrhea, constipation, alopecia, neutropenia, and thrombocytopenia. The DLTs observed were neutropenia and thrombocytopenia that could be due to the bone marrow suppression caused by the combination of C and G alone [18].

The results of this study indicate that the combination of Mif 300 mg, C AUC2, and G 1000 mg/m^2^ or 800 mg/m^2^ was clinically active in metastatic pretreated BC and OC patients but higher than expected rates of neutropenia lead to further dose reductions in G with the requirement for initiation of growth factor support. No responses were observed at lower doses. Neutropenia is a well-known side effect of carboplatin and gemcitabine; therefore, other chemotherapy backbones in combination with mifepristone should be explored. In addition, the effects of GR antagonism with mifepristone on the hematopoetic system itself have yet to be elucidated fully [19]. Further exploration of molecular targets in distinct but functionally linked cancer cell GR-mediated gene expression pathways could represent a suitable strategy in the treatment of both BC and OC.

While the role of GR expression and downstream gene expression pathways in cancer biology is still under investigation, many in vitro and in vivo models suggest that GR contributes to tumor chemoresistance through GR-driven transcriptional activation of pro-survival signaling pathways that prevent caspase-dependent apoptosis. Therefore, targeting GR in these cancers may have potential therapeutic applications. A randomized phase 2 study of a newer, more selective GR antagonist, relacorilant (150 mg the day before, day of, and day after nab-paclitaxel), combined with nab-paclitaxel 80 mg/m^2^ days 1, 8, and 15 of a 28-day cycle) showed a benefit in the arm receiving intermittent (day before, day of and day after) relacorilant in platinum-resistant ovarian cancer (PROC) (hazard ratio [HR] = 0.66; 95% confidence interval [CI] = 0.44–0.98), duration of response (DOR) (HR = 0.36; 95% CI = 0.16–0.77), and overall survival (OS) (HR = 0.67; 95% CI = 0.43–1.03), with minimal added toxicity compared with nab-paclitaxel monotherapy [20]. Recently, the phase III randomized trial of this combination compared to nab-paclitaxel alone in platinum-resistant ovarian cancer (ROSELLA trial NCT05257408) was completed in 381 women. A statistically meaningful improvement in PFS in the dual therapy compared to nab-paclitaxel monotherapy was observed (HR 0.70, 95% CL 0.54–0.91, median 6.5 v 5.5 months, *P* = 0.008) [21]. Further studies are needed to determine whether tumor cell GR expression and associated GR gene expression activity correlate with response and also, to determine a measure for GR transcriptional activity that can predict the likelihood of benefit from treatment with GR antagonism in an individual patient. The long-term antitumor activity reported for one patient with OC in this trial of pretreated metastatic patients is promising. We will continue to study tumor GR and its transcriptional activity as a prognostic and predictive marker in the diagnosis and treatment of GR-positive malignancies.

## Data Availability

No datasets were generated or analysed during the current study.
